# CircMYBL2 facilitates hepatocellular carcinoma progression by regulating E2F1 expression

**DOI:** 10.32604/or.2024.047524

**Published:** 2024-05-23

**Authors:** JUNZHE YI, BINBIN LI, XIAOMIN YIN, LINGRUI LIU, CAILU SONG, YING ZHAO, MANBO CAI, HAILIN TANG, DONG CHEN, NING LYU

**Affiliations:** 1 State Key Laboratory of Oncology in South China, Guangdong Provincial Clinical Research Center for Cancer, Sun Yat-sen University Cancer Center, Guangzhou, 510060, China; 2 Department of Medical Oncology, The Third People’s Hospital of Yongzhou, Yongzhou, 425000, China; 3 Department of Radiotherapy, The First Affiliated Hospital, Hengyang Medical School, University of South China, Hengyang, 421001, China; 4 Center of Hepato-Pancreato-Biliary Surgery, The First Affiliated Hospital, Sun Yat-sen University, Guangzhou, 510080, China

**Keywords:** Circular RNAs, circMYBL2, miR-1205, E2F1, Hepatocellular carcinoma

## Abstract

Circular RNAs (circRNAs) have been recognized as pivotal regulators in tumorigenesis, yet the biological functions as well as molecular mechanisms of the majority of circRNAs in hepatocellular carcinoma (HCC) remain elusive. We sought to unveil the expression profile and biological role of circMYBL2 in HCC. Initial microarray analyses were conducted to probe the expression profile of circMYBL2 in HCC cells, and qRT‒PCR analysis was then performed in HCC cell lines and tissues, revealing significant upregulation of circMYBL2. Subsequent experiments were conducted to evaluate the biological function of circMYBL2 in HCC progression. Furthermore, bioinformatics analysis, qRT‒PCR analysis, luciferase reporter assays, and western blot analysis were employed to investigate the interplay among circMYBL2, miR-1205, and E2F1. CircMYBL2 was found to exhibit marked upregulation in tumor tissues as well as HCC cell lines. Elevated expression of circMYBL2 increased the proliferation and migration of HCC cells, whereas circMYBL2 knockdown elicited contrasting effects. Mechanistically, our results indicated that circMYBL2 promoted E2F1 expression and facilitated HCC progression by sponging miR-1205. Our findings revealed that circMYBL2 contributed to HCC progression through the circMYBL2/miR-1205/E2F1 axis, suggesting the potential of circMYBL2 as a novel target for HCC treatment or a prognostic biomarker for HCC.

## Introduction

Hepatocellular carcinoma (HCC), ranking as the fourth leading cause of tumor-related mortality globally, constitutes about 80% of all liver cancer cases [1–3]. Annually, approximately 600,000 patients succumb to HCC, primarily due to its proclivity for metastasis [4–6]. Despite the rapid development of tyrosine kinase inhibitors and immunotherapies for HCC in recent years, the survival outcomes of HCC patients remain unsatisfactory [7–9]. Consequently, the identification of treatment targets to elucidate potential carcinogenic mechanisms in HCC and the pursuit of more effective treatments are of paramount importance. To date, several studies have identified genes associated with the carcinogenesis of HCC and relevant regulators that may affect the transcription or translation of these genes [10,11]. However, the underlying molecular mechanisms remain elusive [12–14].

In recent years, circular RNAs (circRNAs), along with long noncoding RNAs and miRNAs, have emerged as the most extensively investigated noncoding RNAs (ncRN-As). CircRNAs, distinguished by a single-stranded, covalently closed structure, constitute a class of noncoding RNAs that exhibit high expression levels in a tissue-specific manner [15–17]. Endogenous circRNAs have been recognized for harboring microRNA (miRNA) binding sites, indicating their potential as proficient miRNA sponges that can modulate gene expression through interactions with miRNAs [18,19]. Notably, recent investigations have unveiled the dysregulation of specific circRNAs across certain types of tumors, including HCC, colorectal cancer, and gastric cancer [20–22]. Accumulating evidence underscores the cancer-promoting effects of circRNAs, as they play a regulatory role in cellular functions through their capacity to sponge miRNAs. For instance, circCDR1 facilitates the proliferation and migration of various tumor cells by blocking the function of miR-7 [23–25]. CircEZH2 has been validated to increase the viability and migration of tumor cells, predicting a poorer prognosis in breast cancer [26]. In multiple malignancies, including colon cancer, gastric cancer, and HCC, the circRNA CDR1 modulates the growth and migration of cancer cells through its interaction with miR-7 [27]. Additionally, CircFAT1 hinders the progression of gastric cancer by sponging miR-548g and has been shown to upregulate the RUNX1 tumor suppressor gene [28]. Despite advancements in circRNA research, the molecular mechanisms and potential functions of numerous circRNAs remain elusive. Investigating the roles of circRNAs in HCC carcinogenesis is beneficial for uncovering the fundamental mechanisms driving tumor progression and identifying potential novel therapeutic targets.

circMYBL2 is a circRNA originating from the gene MYBL2, which exhibits diverse roles across various cancers the cell cycle checkpoint gene MYBL2, exhibits diverse roles across various cancers [29–32]. The protein encoded by the MYBL2 gene functions as an oncoprotein and is often linked to unfavorable patient outcomes [33]. It serves as a key transcription factor for genes related to proliferation. circMYBL2 has been found to be significantly upregulated and function as either a tumor-promoting factor or a tumor-suppressing factor in multiple malignancies, including acute myeloid leukemia and cervical cancer [34]. The initial documentation of circMYBL2’s tumor-promoting effects stemmed from a preclinical study conducted by Sun et al. [29]. This study demonstrated that circMYBL2 regulates FLT3 translation by recruiting PTBP1, thereby promoting the progression of acute myeloid leukemia. In another study, circMYBL2 was identified to upregulate EGFR expression by sponging miR-665 and thereby decreased paclitaxel sensitivity in both paclitaxel-sensitive and paclitaxel-resistant cervical cancer cells, accompanied by an increase in the aggressiveness of the tumor cells [30]. Preliminary evidence also suggests the involvement of circMYBL2 in breast cancer progression and liver metastasis [35]. Despite these findings, the biological function and mechanisms of circMYBL2 in HCC remain unexplored.

In this study, we conducted microarray analyses to evaluate the circRNA expression profiles in selected HCC tissues, and we found that circMYBL2 is significantly upregulated in HCC tissues. The results of subsequent functional experiments further demonstrated that circMYBL2 circMYBL2 played a role in promoting the growth and migration of HCC cells and that knockdown of circMYBL2 reversed this tumor-promoting effect. Additionally, we conducted bioinformatic analyses and subsequent experiments to explore the biological functions and potential mechanisms associated with circMYBL2 in HCC progression. Overall, the findings of this study suggest that circMYBL2 may function as a miRNA sponge, specifically binding to miR-1205, thereby establishing a circMYBL2-miR-1205 axis. This interaction leads to the upregulation of E2F1 expression, ultimately facilitating HCC progression. In summary, our studies suggested that circMYBL2 may could potentially function as a novel therapeutic target or a valuable prognostic biomarker or for HCC.

## Materials and Methods

### Patient samples and ethical authorization

This study was authorized by the Ethics Committee of Sun Yat-sen University Cancer Center in Guangzhou, China (SZR2020-03). Tumor samples and matched adjacent normal tissues were collected from patients with HCC. After acquisition, all tissue samples were rapidly frozen in liquid nitrogen. Written informed consent was obtained from all participants.

### Microarray analysis

The circRNA chip utilized in this study comprises probes targeting approximately 170,340 human circRNAs (Capital Biotech human circRNA Array V2.0). Preparation of HCC samples, including both tumor tissues and matched normal tissues were prepared following standard protocols. Microarray hybridization procedures were subsequently conducted. Upregulated circRNAs were identified using a fold change threshold of ≥1, and data visualization was performed with Java TreeView (Stanford University School of Medicine, Stanford, CA, USA).

### Cell culture and transfection

ATCC provided all cell lines (LO2, MHCC-97H, LM3, HepG2 and Huh-7) for this study. The cells were cultured under standard conditions in DMEM (Corning, NY, USA) and confirmed to be mycoplasma-free. A circMYBL2 overexpression vector was constructed by Hanheng Biotech (Shanghai, China). The construct used for RNAi-mediated knockdown of circMYBL2, the miRNA mimic, and the miRNA inhibitor were synthesized by GenePharma (Shanghai, China). Transfection of cells was carried out by Lipofectamine 3000 (Thermo Fisher Scientific, Waltham, MA, USA).

### RNA extraction and quantitative reverse transcription–PCR (qRT‒PCR)

RNA from tissues and cells was extracted using the RNA-Quick Purification Kit (YiShan Biotech, China). Total RNA (2 µg) was reverse transcribed into cDNA utilizing the PrimeScript™ RT Reagent Kit (Takara Bio, Inc., China). Subsequent qRT‒PCR was conducted with SYBR Green Master Mix (TaKaRa, Shiga, Japan). 2 ΔΔCq method was used to determine the relative expression of all target genes. The normalized target transcript abundances were calculated by adopting GAPDH and U6 as endogenous controls. The sequences of the primers used were as follows: CircMYBL2 forward (5′-3′) GCCTCTCTCTTGTTTGTAACCCC, reverse (5′-3′) TCAGAACGCAGCACCTCCTT; MYBL2 forward (5′-3′) CCGGAGCAGAGGGATAGCA, reverse (5′-3′) CAGTGCGGTTAGGGAAGTGG; E2F1 forward (5′-3′) CCGTGGACTCTTCGGAGAAC, reverse (5′-3′) ATCCCACCTACGGTCTCCTC; GAPDH forward (5′-3′) GAAGGTGAAGGTCGGAGTC, reverse (5′-3′) GAAGATGGTGATGGGATTTC; and U6 forward (5′-3′) CGAGCACAGAATCGCTTCA, reverse (5′-3′) CTCGCTTCGGCAGCACATAT.

### Actinomycin D treatment

Cells were cultured in 6-well plates and incubated with actinomycin D (GlpBio) (2 mg/mL). To assess expression changes by qRT‒PCR analysis, all cells were harvested at 0, 6, 12, 18, and 24 h.

### RNase R treatment

RNA extracted from Huh-7 cells or HepG2 cells was incubated with RNase R (Geneseed Biotech, Guangzhou, China) (37°C). Subsequently, the RNA was reverse transcribed to detect circMYBL2 and eliminate the impact of linear MYBL2 RNA.

### Subcellular fractionation

Subcellular isolation of RNA was conducted with nuclear and cytoplasmic extraction reagents (Thermo Fisher Scientific). qRT-PCR was employed to quantify mRNA expression in both cytoplasmic and nuclear fractions.

### Fluorescence *in situ* hybridization (FISH) assay

The FISH assay was executed with a FISH kit obtained from GenePharma (Shanghai, China), and specific probes for circMYBL2 were synthesized by GenePharma (Shanghai, China). To permeabilize HCC cells, 4% paraformaldehyde was applied for 20 min, followed by 0.5% Triton X-100 for 20 min, and the cells were incubated with a circMYBL2 probe overnight.

### Cell counting Kit-8 (CCK-8) assay

Cell proliferation was evaluated using a Cell Counting Kit-8 (CCK-8, GlpBio, USA). Transfected cells were plated and incubated for 24 h (37°C). Then, CCK-8 solution was added, and the plates were incubated for 2 h (37°C). The absorbance at 490 nm was measured by a spectrophotometer (Synergy4; BioTek, Winooski, VT, USA).

### Colony formation assay

Briefly, cells (2 × 10^3^) were seeded in plates to be incubated for 2 weeks (37°C). After fixation in methanol and subsequent staining with 0.1% crystal violet, colonies were enumerated, and the data were analyzed.

### Wound healing assay

Transfected HCC cells were seeded in 6-well plates. The wound healing process was observed using an inverted microscope at both 0 and 24 h after wounding.

### Transwell assay

In the upper chamber, transfected HCC cells were plated in serum-free medium (200 μL), while in the lower chamber, medium supplemented with 20% FBS was added as a chemoattractant. The cells were incubated for 24 h, and the migrated cells were imaged and counted.

### Luciferase reporter assay

Luciferase reporter plasmids containing the circMYBL2-WT or circMYBL2-MT sequence were constructed by Umine-bio (Guangzhou, China). To transfect Huh-7 cells or HepG2 cells, luciferase reporter plasmids and miRNAs were added to 96-well plates. Following a 48-h incubation, luciferase activity was quantified by the Yeasen Biotechnology luciferase assay system.

### Western blot analysis

Proteins were extracted, quantified, separated by 10% SDS‒PAGE, and transferred onto polyvinylidene fluoride membranes (Millipore, MA, USA). The membranes were then blocked with 5% skim milk for 1 h (room temperature) before incubation with an anti-E2F1 antibody (1:1000; Gibco, Carlsbad, CA) or anti-GAPDH antibody (1:1000; Gibco, Carlsbad, CA, USA) overnight (4°C). A secondary antibody (1:5000; Gibco, Carlsbad, CA, USA) was utilized and detected by chemiluminescence.

### Immunohistochemistry

Ki67 or E2F1 expression in harvested tumor specimens was assessed through immunohistochemical staining. In brief, the tissues were fixed with 4% paraformaldehyde, embedded, and sliced into 4-mm-thick sections. Afterward, the selected sections were incubated with an anti-Ki67 antibody (1:200 dilution) or an anti-E2F1 antibody (1:200 dilution) overnight (4°C). After incubation, the sections were stained with diaminobenzidine (DAB) and subjected to microscopy.

### Mouse xenograft model

This study adhered to the guidelines for animal care, and all animal experiments received approval from the Institutional Animal Experimentation Ethics Committee of Sun Yat-sen University (L102022020001T). Male BALB/c nude mice (4 weeks old) were inoculated with 2 × 10^6^ cells subcutaneously (6 mice per group). Then, 40 μl of si-circMYBL2 or control siRNA was administered via intratumoral injection every 4 days. At the conclusion of a 4-week period, the mice were euthanized, and the size as well as the weight of the tumors were measured.

### Statistical analysis

All data were analyzed with GraphPad Prism 8.0 (La Jolla, CA, USA). Quantitative data are expressed as the means ± standard deviations (SDs). Group comparisons were performed using a two-tailed Student’s *t-*test or one-way analysis of variance as appropriate. Pearson correlation analysis was employed to assess correlations between two groups, with *p* < 0.05 indicative of statistical significance. Each experiment was performed with three biological replicates, and all representative micrographs shown are from one of three independent experiments.

## Results

### CircMYBL2 was upregulated in HCC tissues and HCC cell line

**Figure 1:**
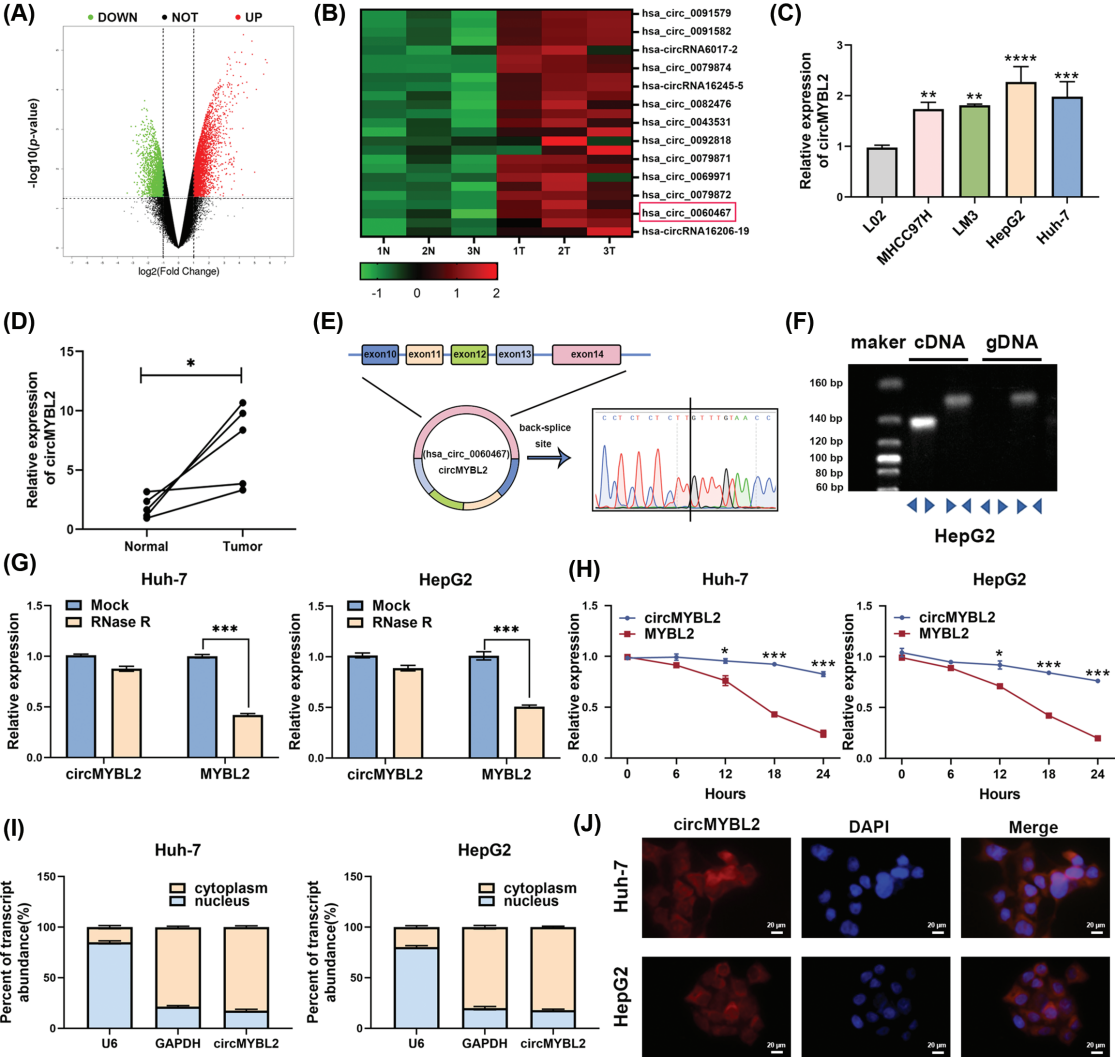
CircMYBL2 exhibits a pronounced upregulation in both HCC cell lines and tissues. (A) Volcano plot showing the circRNA expression in 3 pairs of HCC tissues. The filtering criteria were log2 (fold change) ≥1 and *p* value < 0.05. (B) Heatmap showing the top 25 upregulated circRNAs in HCC tissues in comparison to normal liver tissues. (C) The circMYBL2 expression in the cell line LO2, MHCC97H, LM3, HepG2 and Huh-7 was measured by qRT‒PCR. (D) The expression of circMYBL2 in in an additional set of five paired tissues, including HCC tissues and corresponding nontumor tissues was measured by qRT‒PCR. (E) Schematic representation of circMYBL2 formation through the circularization of exons in the MYBL2 gene. (F) PCR analysis utilizing both divergent and convergent primers demonstrated the amplification of circRNAs from gDNA or cDNA of HepG2 HCC cells. (G) The expression of circMYBL2 and the linear MYBL2 in HCC cells incubated with Mock or RNase R. (H) In HCC cell lines treated with actinomycin D, the expression of circMYBL2 and the corresponding MYBL2 mRNA was assessed. (I) Subcellular fractionation assay results showing the expression of circMYBL2 in both the cytoplasm and nucleus of HCC cells. U6 (nuclear control transcript), GAPDH (cytoplasmic control transcript) and circMYBL2 levels are shown. Normalization of CircMYBL2 expression was conducted by aligning it with GAPDH expression in the cytoplasm and U6 expression in the nucleus. (J) The subcellular localization of circMYBL2 was visualized by RNA FISH. Nuclei were stained with DAPI. *p < 0.05; **p < 0.01; ***p < 0.001; ****p < 0.0001. FISH, fluorescence *in situ* hybridization. Each experiment was independently repeated three times.

To identify differentially expressed circRNAs, microarray analyses were performed in 3 pairs of HCC tissues and matched normal tissues (Fig. 1A). The heatmap illustrates the top 25 upregulated circRNAs, with hsa_circ_0060467, termed circMYBL2, emerging as the most significantly upregulated in the selected samples (Fig. 1B). Therefore, we evaluated the expression of circMYBL2 among the human normal liver cell line LO2 and 4 other HCC cell lines, and statistical analysis revealed a significant increase in the circMYBL2 level in HCC cells (Fig. 1C). Two HCC cell lines, Huh-7 and HepG2, were selected for subsequent cell experiments. The expression of circMYBL2 was further evaluated in an additional 5 pairs of normal tissues and primary HCC tissues, and significantly increased expression of circMYBL2 was observed (*p* < 0.05), consistent with the above results (Fig. 1D).

To validate the formation of circMYBL2, its head-to-tail splicing was detected by Sanger sequencing (Fig. 1E). Divergent primers were used to amplify circMYBL2 circular transcripts, while convergent primers were employed for amplification of MYBL2 linear transcripts. The results of PCR analysis indicated that the divergent primers amplified circMYBL2 from cDNA, while the convergent primers amplified MYBL2 from both gDNA and cDNA (Fig. 1F). To provide further confirmation of the circular structure of circMYBL2, we conducted an RNase R digestion assay, and the results demonstrated that circMYBL2 exhibited higher resistance to RNase R digestion than linear MYBL2 (Fig. 1G). In addition, we treated Huh-7 as well as HepG2 cells with actinomycin D to evaluate the stability of circMYBL2, and the results suggested that circMYBL2 was more stable and had a longer half-life than linear MYBL2 (Fig. 1H). Subsequently, subcellular fractionation and FISH assays were utilized to measure circMYBL2 expression in HCC cells, and our observations revealed predominant expression of circMYBL2 in the cytoplasm (Figs. 1I and 1J).

### CircMYB2 promoted hepatocellular carcinoma progression

**Figure 2:**
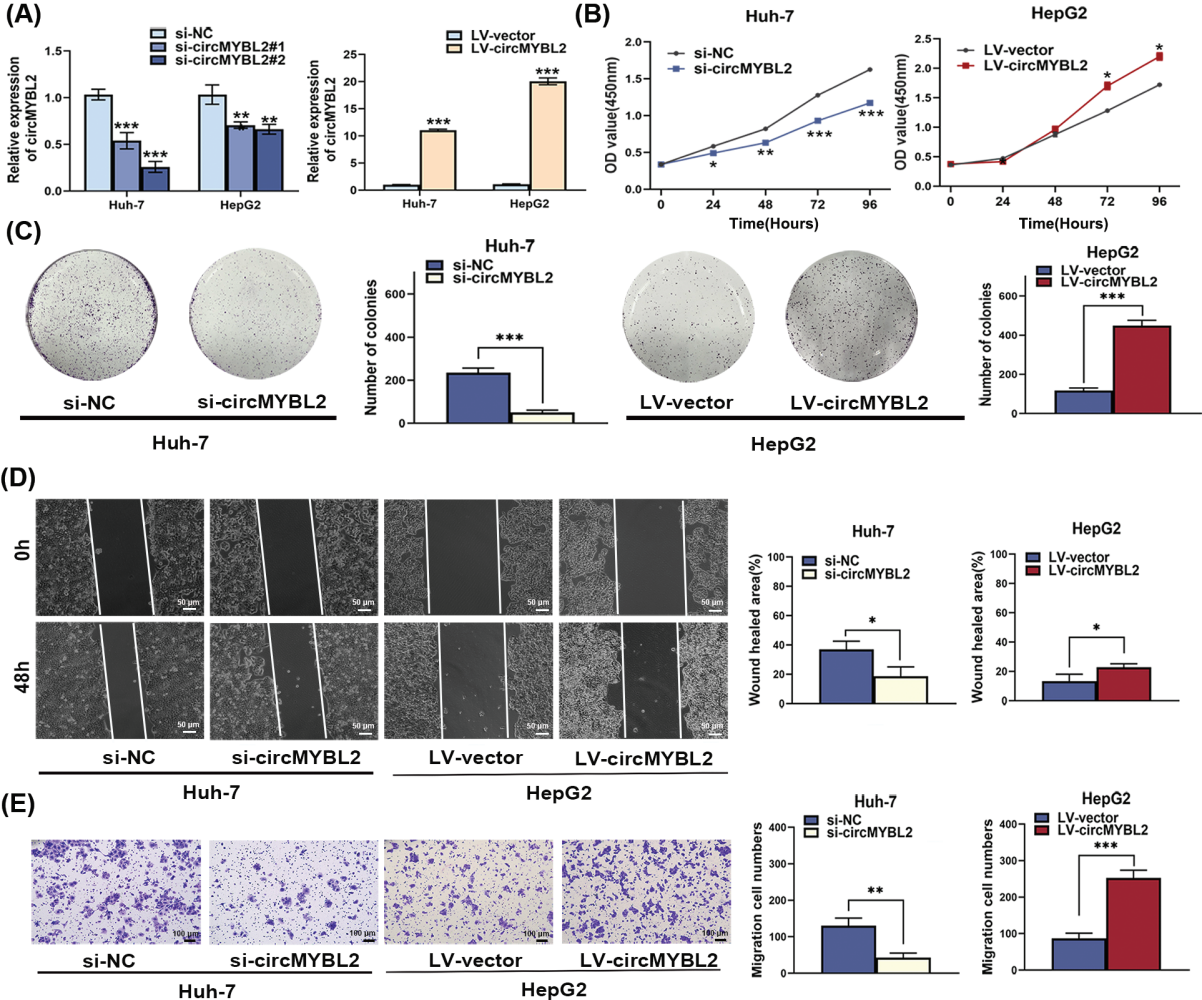
CircMYB2 promoted the proliferation and migration of HCC cells *in vitro*. (A) Successful knockdown of circMYBL2 with si-circMYBL2 and overexpression of circMYBL2 in HCC cells transduced with LV-circMYBL2. (B) Cell proliferation was assessed in Huh-7 and HepG2 cells. (C) Overexpression of circMYBL2 resulted in an augmented colony-forming ability in HepG2 cells, whereas the knockdown of circMYBL2 led to a reduced colony-forming ability in Huh-7 cells. (D) The impact of circMYBL2 on the cell migration capability was assessed by a wound healing assay was performed to assess. The scale bar in the images of the wound healing assay denotes a length of 50 μm. (E) To assess the influence of circMYBL2 on migration ability, a Transwell assay was employed. The scale bar in the Transwell assay images is indicative of 100 μm. *p < 0.05; **p < 0.01; ***p < 0.001. LV, lentiviral vector; NC, negative control; OD, optical density. Each experiment was independently repeated three times.

**Figure 3:**
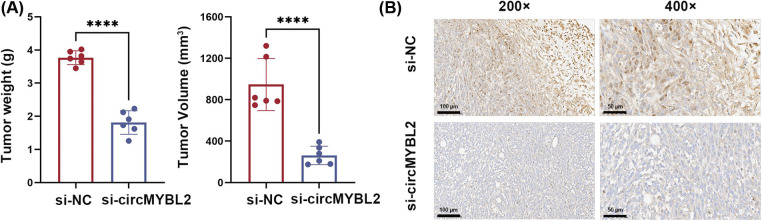
Silencing circMYBL2 contributes to the suppression of tumor growth *in vivo*. (A) The weight and volume measurements of the xenograft tumors were recorded. (B) Immunohistochemical sections showing the expression of Ki67 in harvested xenograft tumors treated with si-circMYBL2 or control siRNA are shown. The scale bar in the immunohistochemical images is indicative of 100 μm (200×) and 50 μm (400×), respectively, ****p < 0.0001. NC, negative control. Each experiment was independently repeated three times.

To explore the functions of circMYBL2 in HCC development, two siRNAs were utilized to knock down circMYBL2. The knockdown efficiency of each siRNA was verified by measuring the circMYBL2 level in the two cell lines (Fig. 2A). In addition, we designed lentiviral vectors to stably overexpress circMYBL2 in HCC cells (Fig. 2A). The results of CCK-8 and colony formation assays showed that the downregulation of circMYBL2 resulted in a notable suppression of HCC cells. Conversely, the overexpression of circMYBL2 demonstrated a stimulatory effect on HCC cell proliferation (Figs. 2B and 2C). Subsequently, to assess the function of circMYBL2 in HCC cell migration, wound healing and Transwell assays were carried out. Downregulation of circMYBL2 notably suppressed the migration of Huh-7 and HepG2 cells, and an increased migration capability was observed after overexpression of circMYBL2 (Figs. 2D and 2E). Next, mouse xenograft experiments were carried out to further evaluate the function of circMYBL2 in tumor growth *in vivo*. In line with the abovementioned results from the *in vitro* experiments, circMYBL2 knockdown significantly decreased tumor growth (Figs. 3A and 3B). Additionally, immunohistochemical staining of harvested xenograft tumors indicated that circMYBL2 knockdown decreased the Ki-67 expression level. Collectively, these findings indicated that downregulation of circMYBL2 significantly reduced the malignancy of HCC cells, underscoring its oncogenic function in HCC.

### CircMYBL2 served as a sponge of miR-1205

**Figure 4:**
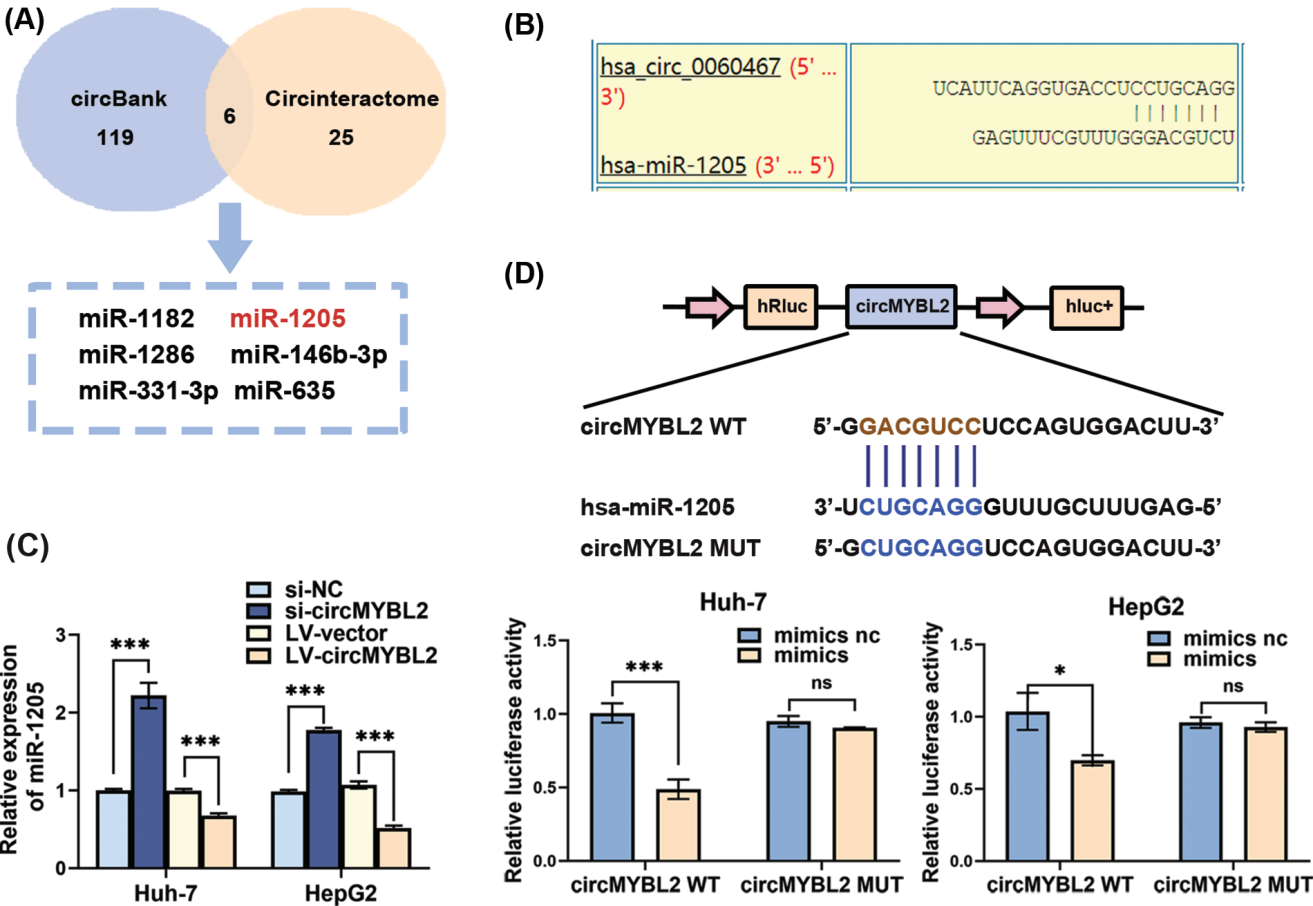
CircMYBL2 served as a miRNA sponge of miR-1205. (A) A Venn diagram showing the overlapping candidate binding miRNAs associated with circMYBL2, derived from both the circBank and CircInteractome databases. (B) The predicted binding sites of miR-1205 within the circMYBL2 sequence based on CircInteractome databases. (C) miR-1205 expression was measured by qRT‒PCR in HCC cells incubated with si-circMYBL2 or LV-circMYBL2. (D) Relative luciferase activity in HCC cells cotransfected with a luciferase reporter vector containing the wild-type or mutated circMYBL2 sequence and the miR-1205 mimic. All presented data are represented as the mean ± SD. Statistical significance is denoted as follows: *p < 0.05; ***p < 0.001. NC, negative control; WT, wild-type; MUT, mutant. Each experiment was independently repeated three times.

Given the established role of circRNAs as miRNA sponges, thereby neutralizing the functions of relevant miRNAs in multiple types of cancers [36,37], we employed the circBank and CircInteractome databases to predict candidate binding miRNAs of circMYBL2. Subsequently, we identified six miRNAs through preliminary screening (Fig. 4A). We measured the expression of these six miRNAs in HCC cells by qRT‒PCR and observed a negative correlation between miR-1205 and circMYBL2. We further identified the potential binding site of miR-1205 within the circMYBL2 sequence (Fig. 4B). The qPCR analysis results showed that miR-1205 was upregulated with circMYBL2 knockdown but downregulated with overexpression of circMYBL2 in HCC cells (Fig. 4C). To confirm the anticipated binding interaction, we executed a luciferase reporter assay to evaluate the direct association between circMYBL2 and miR-1205. Transfection of cells with the wild-type luciferase reporter resulted in diminished luciferase activity upon miR-1205 mimic transfection. However, this effect was not observed in cells transfected with the mutant luciferase reporter, indicating a potential role of circMYBL2 as a miR-1205 sponge in HCC cells (Fig. 4D).

### CircMYBL2 promoted HCC development via the circMYBL2-miR-1205-E2F1 axis

**Figure 5:**
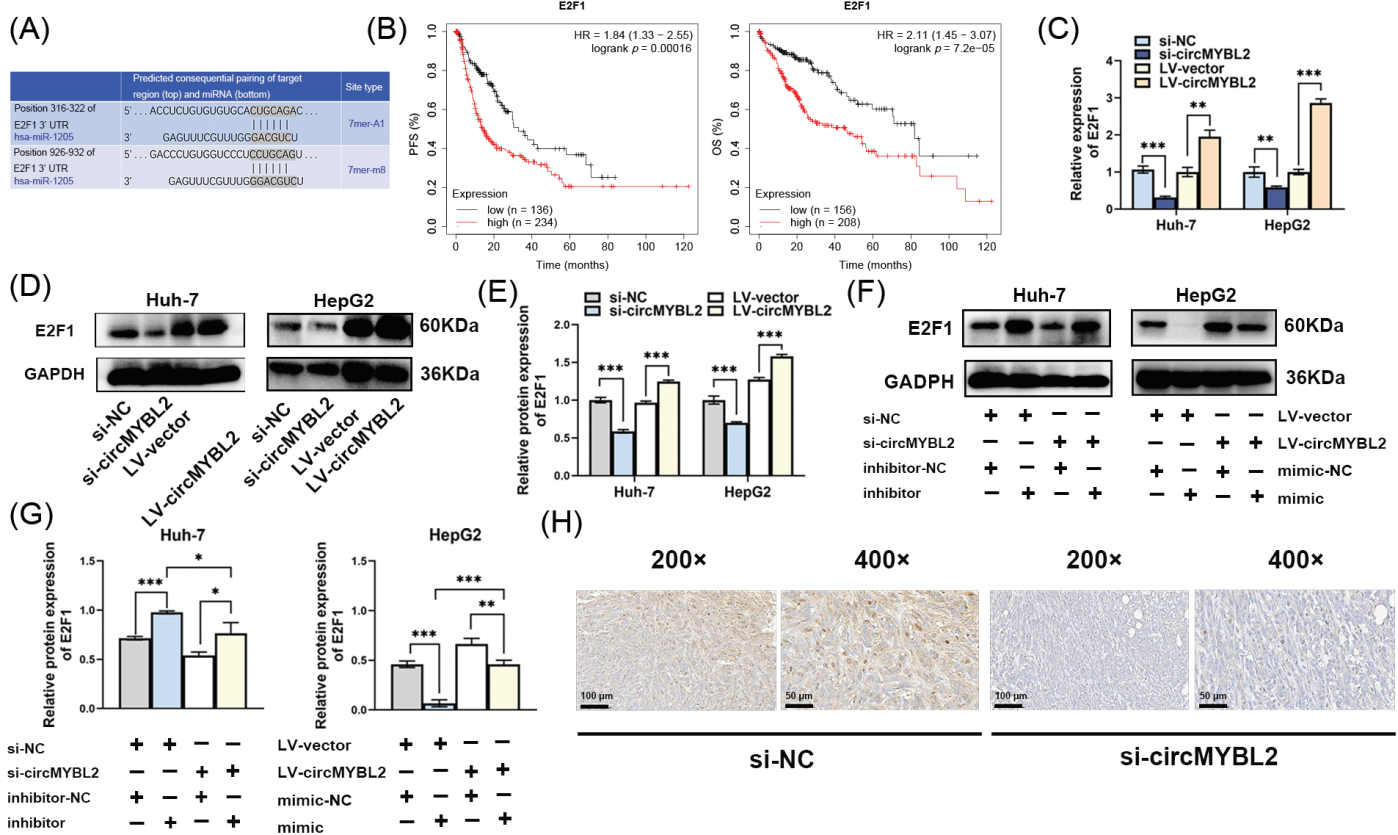
CircMYBL2 promotes the proliferation and migration of HCC cells through the circMYBL2-miR-1205-E2F1 axis. (A) The TargetScan algorithm predicted the presence of binding sites of miR-1205 within the 3′-UTR of E2F1. (B) Kaplan‒Meier analysis of the associations between E2F1 expression and progression-free survival and overall survival of HCC patients (http://kmplot.com/analysis). (C–E) The expression levels of miR-1205 were quantified through both qRT‒PCR and western blotting in HCC cells subjected to incubation with si-circMYBL2 or LV-circMYBL2. (F, G) Huh-7 cells were cotransfected with si-circMYBL2/si-NC and the miR-1205 inhibitor/inhibitor-NC, and HepG2 cells were transduced with LV-circMYBL2/LV-vector and transfected with the miR-1205 mimic/mimic-NC. The impact of circMYBL2 silencing on the protein expression of E2F1, as determined by western blot analysis, was reversed after miR-1205 silencing. (H) Expression status of E2F1 in immunohistochemical sections of harvested xenograft tumors. The scale bar in the immunohistochemical images is indicative of 100 μm (200×) and 50 μm (400×), respectively, *p < 0.05; **p < 0.01; ***p < 0.001. LV, lentiviral vector; NC, negative control. Each experiment was independently repeated three times.

To investigate the downstream targets regulated by miR-1205, we used the TargetScan algorithm and identified E2F1 as a potential target oncogene (Fig. 5A), an identity that has been confirmed by existing research evidence [38–41]. We also found that elevated expression of E2F1 correlated with short progression-free survival and overall survival times in HCC patients via analysis of a public database (Fig. 5B). qRT‒PCR analysis revealed that E2F1 was downregulated in HCC cells transfected with siRNAs targeting circMYBL2 but was upregulated in HCC cells also transduced with the lentiviral circMYBL2 overexpression vector (LV-circMYBL2) (Fig. 5C). E2F1 expression, as measured by western blot analysis, was significantly decreased when circMYBL2 was knocked down and increased when HCC cells were transduced with LV-circMYBL2 (Figs. 5D and 5E). To further verify the hypothesis suggesting a regulatory axis involving circMYBL2, miR-1205, and E2F1, HCC cells were transfected with si-MYBL2/the miR-1205 inhibitor or LV-circMYBL2/the miR-1205 mimic, and E2F1 expression was analyzed by western blotting (Fig. 5F). Taken together, the experimental results revealed that knocking down circMYBL2 downregulated the expression of E2F1 and that transfection of the miR-1205 inhibitor reversed this change in expression to some extent. We observed the opposite results in the groups of HCC cells transduced with LV-circMYBL2 and/or transfected with the miR-1205 mimic (Fig. 5G). In addition, it was further confirmed that knockdown of circMYBL2 decreased the E2F1 expression level in immunohistochemical sections of harvested xenograft tumors (Fig. 5H). Collectively, the above results suggest that circMYBL2 serves as a competing endogenous RNA to increase E2F1 expression by sponging miR-1205.

## Discussion

Recently, researchers have increasingly focused on the roles of circRNAs in carcinogenesis. Given the unclear function and expression profile of many circRNAs in HCC progression and considering the prevalent functional paradigm of circRNAs as miRNA sponges [42], we conducted chip microarray analyses and aimed to explore the regulatory role and potential molecular mechanisms of circMYBL2 overexpression in HCC. In our study, we explored the expression profile of circRNAs and noted a substantial upregulation of circMYBL2 in both HCC cell lines and tissues. CircMYBL2 has been implicated in various malignancies, including myeloid leukemia, cervical cancer, and pancreatic adenocarcinoma [29–32]. A previous study also identified its involvement in the process of cell proliferation and epithelial-mesenchymal transition, thus contributing to breast cancer progression and liver metastasis [35]. The involvement of circMYBL2 in the tumorigenic behaviors and aggressiveness of liver-derived tumor cells remains unclear. To address this uncertainty, we conducted a series of functional experiments, revealing that circMYBL2 knockdown inhibited HCC cell proliferation and migration, while circMYBL2 overexpression had the opposite effects. Despite the limited number of selected HCC tissue samples, our data suggested the tumor-promoting effects of circMYBL2 in HCC. However, the potential involvement of other crucial deregulated circRNAs in HCC development cannot be excluded. Further exploration of the roles of circRNAs in HCC progression is warranted, and we will continue to focus on this issue in our future research.

In this study, we confirmed by bioinformatics analysis and subsequent experiments, that the circMYBL2 sequence contained the miRNA response elements of miR-1205 and acted as a molecular sponge of miR-1205. miRNAs are short double-stranded RNAs recognized as crucial regulators of cell growth, adhesion, differentiation, and migration, and they play well-established roles in various tumors, including HCC [43]. Several studies have highlighted the significant involvement of miR-1205 in various tumor types. For example, miR-1205 has been demonstrated to suppress the migration and growth of gastric cancer cells through its interaction with circCYFIP2 [40]. Wang et al. demonstrated that miR-1205 accelerates cell proliferation in prostate cancer [44]. Additionally, Yang et al. revealed that circ_0034642 increases the proliferation and invasion of glioma cells by sponging miR-1205, which itself may function as a tumor suppressor [45]. Our results further support this trend by showing that circMYBL2 downregulates miR-1205 expression in HCC, indicating that the circMYBL2miR-1205 interaction may play a regulatory role in HCC progression.

E2F1 is a well-established transcription factor involved in modulating essential cellular processes [46–48]. Studies conducted previously have illustrated that E2F1 functions as a downstream target of miR-1205, providing further substantiation for the modulatory effects of the miR-1205/E2F1 signaling pathway on tumor progression [38–41]. A recent study indicated a downregulation of miR-1205 in laryngeal carcinoma tissue and that this downregulation could be counteracted when E2F1 was overexpressed [38]. Remarkably, a recent investigation exploring the mechanism of sorafenib resistance revealed that circFN1 knockdown partly sensitized HCC cells to sorafenib by modulating miRNA-1205 and E2F1 expression [41]. These findings suggest that miR-1205 binds to the 3′-UTR of E2F1, leading to E2F1 downregulation. Moreover, the observed overexpression of E2F1 in human HCC tissues compared to adjacent normal specimens further supports its potential role in HCC [49]. In line with these previous studies, our investigation revealed that both the 3′-UTR of E2F1 and circMYBL2 contain miR-1205 binding sites. Furthermore, heightened expression of E2F1 exhibited a correlation with unfavorable survival outcomes in HCC patients. Taken together, these observations support the hypothesis that miR-1205 might regulate HCC progression by targeting E2F1, an idea further validated through western blot analysis in this study. Recently, several studies have revealed the pro-proliferative role of E2F1 in HCC cells. For instance, Simile et al. performed experiments such as cell counting assays, viability assays, microscopic observation and cell cycle analysis in three HCC cell lines, namely, Huh 7, HepG2, and JHH6, and reported that siRNA-mediated depletion of E2F1 suppressed cell growth [50]. In addition to identifying the regulation of the proliferation of HCC cells by E2F1, related research has also revealed that E2F1 triggers apoptosis by activating apoptotic genes. The proapoptotic function of E2F1 was observed in HCC cell lines overexpressing E2F1 [51]. Collectively, the current evidence suggests that E2F1 has a dual function in promoting proliferation and apoptosis in HCC cells, and the proliferative effect seems to be more pronounced [47]. In the early stage of tumors, E2F1 promotes both cell proliferation and apoptosis and has been shown to delay tumor progression. In more advanced stages, the proapoptotic function is gradually lost, and thus the proliferative effect of E2F1 becomes dominant [52,53]. Consistent with previous studies, the bioinformatic analyses in our study revealed that both the 3′-UTR of E2F1 and circMYBL2 contain miR-1205 binding sites, and western blotting further indicated that circMYBL2 regulates E2F1 expression by sponging miR-1205.

In conclusion, this study revealed significant upregulation of circMYBL2 in both HCC tissues and HCC cells. The identified mechanism demonstrated that circMYBL2 modulates the expression of E2F1 by sponging miR-1205, consequently promoting the proliferation and migration of HCC cells. Consequently, circMYBL2 emerges as a promising therapeutic target for HCC treatment and as a prognostic biomarker for HCC.

## Data Availability

The analyzed datasets generated during the study are available from the corresponding author on reasonable request.
